# High-Dose Tranexamic Acid Enhances Circulating Neutrophil Extracellular Traps and Thrombus in Thrombosis Mouse Model

**DOI:** 10.3390/biomedicines13061284

**Published:** 2025-05-23

**Authors:** Jung-Wook Song, Eun-Hye Seo, Un Yung Choi, Chung-Sik Oh, Aram Kim, Keeho Song, Seung-Hyun Lee, Jin Kook Kim

**Affiliations:** 1 Department of Infection and Immunology, Konkuk University School of Medicine, Seoul 05030, Republic of Korea; wook8215@naver.com (J.-W.S.); shlee@kku.ac.kr (S.-H.L.); 2 Korea mRNA Vaccine Initiative, Gachon University, Seongnam 13120, Republic of Korea; gmreo@naver.com; 3 Department of Microbiology, Konkuk University School of Medicine, Chungju 27478, Republic of Korea; uychoi@kku.ac.kr; 4 Research Institute of Medical Science, Konkuk University School of Medicine, Seoul 05030, Republic of Korea; 5 Department of Anesthesiology and Pain Medicine, Konkuk University Medical Center, Konkuk University School of Medicine, Seoul 05030, Republic of Korea; 6 Institution of Biomedical Sciences and Technology, Konkuk University School of Medicine, Seoul 05030, Republic of Korea; 7 Department of Urology, KonKuk University Medical Center, KonKuk University School of Medicine, Seoul 05030, Republic of Korea; arkim@kuh.ac.kr; 8 Division of Endocrinology and Metabolism, Department of Internal Medicine, Konkuk University Medical Center, Konkuk University School of Medicine, Seoul 05030, Republic of Korea; skh2k@kuh.ac.kr; 9 Department of Otorhinolaryngology-Head and Neck Surgery, Konkuk University Medical Center, Konkuk University School of Medicine, Seoul 05030, Republic of Korea; entalk@kuh.ac.kr

**Keywords:** thrombosis, tranexamic acid, neutrophil, platelet, endothelium

## Abstract

**Background/Objectives**: Tranexamic acid (TXA) reduces mortality in patients with massive hemorrhage by inhibiting fibrinolysis. However, it is associated with an increased risk of thrombosis. The activation of neutrophil extracellular traps (NETs) has been implicated in the formation of thrombosis. This study investigated the effects of tranexamic acid on circulating and localized NETs, neutrophils, platelets, and the vascular endothelium in a mouse model of thrombosis. **Methods**: A ferric chloride-induced thrombosis mouse model was used and divided into five groups: a Control group that received intraperitoneal phosphate-buffered saline (PBS), and four experimental groups that received intraperitoneal tranexamic acid at doses of 5 mg/kg, 10 mg/kg, 20 mg/kg, and 30 mg/kg, respectively. To evaluate the expression of circulating and localized NETs, neutrophils, platelets, vascular endothelial cells, fibrinogen, and D-dimer, the following markers were analyzed: myeloperoxidase (MPO), neutrophil marker, cluster of differentiation (CD)31, CD34, fibrinogen α-chain, and D-dimer. These markers were assessed using flow cytometry, immunohistofluorescence staining, and Western blot analysis. The primary endpoint was the differential expression of anti-MPO antibody among the groups. **Results**: In total, data from 20 thrombosis mouse models were analyzed. For each group, four samples were assessed by flow cytometry, and three samples by immunohistofluorescence staining and Western blot analysis, respectively. In the flow cytometric analysis, circulating anti-MPO antibody expression was significantly higher in the TXA 20 and TXA 30 groups compared to the Control group (*p* = 0.001 and *p* = 0.001, respectively). Immunohistofluorescence staining revealed that D-dimer expression in the thrombotic femoral artery was significantly lower in the TXA 5, TXA 10, and TXA 20 groups compared to the Control group (*p* = 0.005; *p* = 0.018; *p* = 0.004, respectively), but significantly higher in the TXA 30 group than in the Control group (*p* = 0.044). Similarly, the expression of anti-fibrinogen antibody was significantly lower in the TXA 5, TXA 10, and TXA 20 groups compared to the Control group (*p* = 0.038; *p* = 0.003; *p* = 0.041, respectively). Western blot analysis showed no significant differences in the expression of anti-Ly6B.2, anti-fibrinogen, and anti-CD31 antibodies among the groups. **Conclusions**: The present study suggests that high-dose tranexamic acid (30 mg/kg) administration may increase circulating NETs and localized D-dimer levels, indicating a higher potential for thrombosis in a thrombosis mouse model. These findings imply that the prothrombotic effects of tranexamic acid may be dose-dependent and could vary based on underlying disease conditions. Therefore, the careful dosage adjustment of tranexamic acid may be necessary, particularly in patients at risk of thrombosis.

## 1. Introduction

Thrombosis is a highly prevalent condition worldwide and remains a leading cause of mortality [1]. It typically occurs as a result of vascular wall injury, which triggers acute platelet activation and the formation of a platelet plug. This is followed by the activation of coagulation factors and the development of fibrin-rich thrombi at the site of injury [2]. Upon vascular damage, platelets rapidly aggregate at the site, initiating a cascade in which prothrombin is converted into thrombin. Thrombin then catalyzes the conversion of fibrinogen into fibrin. The resulting fibrin mesh stabilizes the platelet plug and interacts with other coagulation factors, ultimately leading to thrombus formation through the coagulation cascade [3,4].

Hemostatic agents, which function in contrast to anticoagulants, are designed to reduce bleeding time and limit blood loss. These agents are commonly administered before or after injury to promote hemostasis [5]. Tranexamic acid (TXA) is an antifibrinolytic agent that inhibits fibrinolysis by competitively binding to the lysine-binding sites of plasminogen [6]. This prevents plasminogen from binding to fibrin and inhibits its conversion to plasmin. Consequently, TXA stabilizes clot formation by preventing the degradation of fibrin and other coagulation components within the thrombus, thereby enhancing hemostasis [7,8]. Previous guidelines have suggested that TXA doses ranging from 10 mg/kg to 20 mg/kg are generally considered safe with respect to thrombotic risk [1,9]. However, the potential for TXA to increase thrombotic events remains a topic of ongoing debate [10,11], and its effects on thrombus formation, particularly in individuals with a predisposition to thrombosis, are not yet fully understood [12].

Leukocyte activation plays a crucial role in the inflammatory responses associated with thrombus formation and cardiovascular diseases [13]. Among the leukocyte subtypes, neutrophils are key contributors to both arterial and venous thrombosis, in part by modulating the hemostatic functions of endothelial cells and platelets [14]. Activated neutrophils have been linked to thrombus formation in patients with deep vein thrombosis [15]. A critical mechanism by which neutrophils contribute to thrombosis is through the release of neutrophil extracellular traps (NETs), which serve as structural components of thrombi. NETs bind fibrinogen and promote fibrin formation [16], while also exerting antifibrinolytic effects by inhibiting anticoagulation pathways [17]. NETs have been strongly associated with thrombus formation in patients with deep vein thrombosis [18]. These findings raise the possibility that TXA administration, particularly at higher doses, may interact with NETs to enhance thrombus formation under prothrombotic conditions. However, few studies have investigated the potential association or synergistic antifibrinolytic effects between NETs and TXA at doses exceeding 20 mg/kg in the context of thrombosis.

Therefore, we hypothesized that thrombus formation would be affected by the dose of TXA and would have a relationship with NETs in thrombotic circumstances. This study was designed to investigate the effects of TXA dose on thrombus formation in a thrombosis mouse model.

## 2. Materials and Methods

This study was approved by the Institutional Animal Care and Use Committee (IACUC) of Konkuk University (approval number: KU23002-1), in accordance with the National Institutes of Health (NIH) guidelines for the care and use of laboratory animals. All animal experiments were performed at the Laboratory Animal Center of the Department of Microbiology, Konkuk University School of Medicine, under the management and supervision of the Konkuk University IACUC guidelines. To ensure consistent results, all surgical procedures in the present study were conducted by the same researcher.

### 2.1. Preparation of Thrombosis Mouse Model with TXA Administration

A ferric chloride (FeCl_3_)-induced thrombosis mouse model was employed in this study, as it is widely used to evaluate procoagulant effects and is well regarded for its high reproducibility [19,20,21]. The carotid artery is a commonly used model for arterial thrombosis studies due to its size and relevance to stroke and cardiovascular research, but it has limitations such as lower surgical accessibility, and in this study, blood flow was not measured [22]. Alternatively, the femoral artery is frequently used for investigating general thrombus formation mechanisms and antithrombotic agents, offering easier surgical access and versatility in thrombosis induction methods [23]. Accordingly, we selected the femoral artery as the target vessel and applied the same thrombosis induction technique as used in the carotid artery model.

A total of 20 male C57BL/6NRj mice were randomly assigned to five groups (Control, TXA 5, TXA 10, TXA 20, and TXA 30) with 4 mice per group. Prior to the induction of thrombosis with 2.5% FeCl_3_, the mice were anesthetized using an inhalation anesthesia system (Harvard Apparatus, Holliston, MA, USA). Anesthesia induction was performed by placing each mouse in an induction chamber containing 5.0 vol% sevoflurane with oxygen for 1 min. Following induction, anesthesia was maintained using a nose cone delivering 2.0 vol% sevoflurane with oxygen to ensure that the animals remained unconscious throughout the procedure. Body temperature was continuously maintained using a heating pad. The femoral region was selected for its accessibility and suitability for evaluating drug-induced thrombosis. Following the shaving of the area, a skin incision was made using a surgical blade, followed by the careful dissection of the underlying soft tissue to expose the femoral artery and vein. The surrounding tissues were gently separated to ensure a clear view of the femoral artery for subsequent FeCl_3_ application. Filter paper strips (30 mm × 5 mm) were thoroughly soaked in a 2.5% FeCl_3_ solution for approximately 20 min prior to application. The saturated filter papers were placed directly onto the exposed femoral artery and vein for 30 min, with replacements every 10 min to prevent drying and ensure consistent FeCl_3_ exposure. Within 1 min following the first application of FeCl_3_-saturated filter paper to the femoral artery, the Control group received an intraperitoneal injection of phosphate-buffered saline (PBS), while the TXA 5, TXA 10, TXA 20, and TXA 30 groups received intraperitoneal injections of tranexamic acid (TXA) at doses of 5 mg/kg, 10 mg/kg, 20 mg/kg, and 30 mg/kg, respectively. This protocol was designed to ensure that TXA and PBS were administered at the same time point across all groups. Allometric scaling, a method that accounts for interspecies differences in body surface area relative to body weight and applies species-specific safety factors, was employed to determine the appropriate TXA dosage for the cross-species translation of therapeutic dosing [24]. Following treatment and FeCl_3_ application, blood samples were collected from all groups to evaluate and compare thrombus formation. Subsequently, the femoral artery was carefully dissected, harvested, and processed for downstream tissue analyses.

### 2.2. Blood Collection and Peripheral Blood Mononuclear Cell (PBMC) Isolation

Following the thoracic incision, approximately 800 µL of blood was collected via cardiac puncture from four samples in each group. No anticoagulant was added to the collected blood samples from either the Control or TXA-treated groups. Instead, all samples were immediately diluted with Dulbecco’s phosphate-buffered saline (DPBS; Cytiva, Marlborough, MA, USA). To evaluate thrombus formation, peripheral blood mononuclear cells (PBMCs) were isolated from whole blood in both the Control and TXA-treated groups, and thrombotic markers were analyzed and compared. Specifically, each blood sample was diluted at a 1:5 ratio with DPBS. Ficoll-Paque (Cytiva, USA) was gently added to the bottom of a 15 mL conical tube at a 1:2 ratio relative to the diluted blood volume. The diluted blood was then gently layered on top of the Ficoll-Paque solution, taking care not to disturb the interface. The total volume of the diluted blood and Ficoll-Paque mixture was adjusted to 7 mL. Centrifugation was performed at 1000× *g* for 10 min with the brake turned off. Following centrifugation, the plasma and PBMC layer (white cloudy layer) just above the Ficoll interface was carefully collected using a transfer pipette and transferred to a new 15 mL conical tube. The collected fraction was washed with 5 mL DPBS and centrifuged at 1600 rpm for 10 min. The supernatant was discarded, and the cell pellet was retained. To remove residual red blood cells (RBCs), the pellet was resuspended in 300 µL of RBC lysis buffer (Invitrogen, Waltham, MA, USA) and incubated at room temperature for 10 min. Samples were then centrifuged at 1600 rpm for 10 min, and the supernatant was discarded. The washing step was repeated at least twice with 5 mL of DPBS each time. After the final wash, the PBMC pellet was resuspended in PBS. A small aliquot of the PBMC suspension was stained for cell counting. The desired number of cells was then calculated, and samples were transferred into 15 mL polystyrene conical tubes for subsequent flow cytometric analysis.

### 2.3. Isolation of Femoral Artery

Femoral arteries were harvested from the first three mice out of a total of four in each group for analysis. The artery from the remaining mouse was not collected due to its small diameter, which made it impossible to obtain an adequate amount of sample. The femoral artery was carefully isolated and longitudinally sectioned into three equal proximal, middle, and distal segments. The proximal segment of the sectioned femoral artery was stored at −20 °C [25,26] in a 5 mL conical tube for protein analysis, while the distal segment was placed into a 5 mL conical tube for subsequent flow cytometric analysis. The middle segment of the femoral artery was fixed in 4% paraformaldehyde, placed in a 5 mL conical tube, and stored at 4 °C for subsequent histological and immunofluorescence analyses.

### 2.4. Circulating NETs and Neutrophils Assessed by Flow Cytometry

For flow cytometric analysis, PBMCs isolated from whole blood were transferred into 15 mL polystyrene conical tubes and fixed with 300 µL of Cytofix fixation buffer (BD Biosciences, San Diego, CA, USA) for 15 min at room temperature. Following fixation, 5 mL of cold DPBS was added, and the cells were gently resuspended. The fixed cells were then permeabilized using 1× Perm/Wash buffer (BD Biosciences, USA) and centrifuged at 1600 rpm for 10 min.

To detect NETs and neutrophils, anti-myeloperoxidase (MPO) antibody (Abcam, Cambridge, UK), targeting the MPO light chain as a marker of NETs, and anti-neutrophil (Ly6B.2) antibody (Abcam, UK), targeting the Ly-6B.2 antigen as a neutrophil marker, were used as primary antibodies. Both antibodies were diluted 1:500 in staining buffer (BD Biosciences, USA) supplemented with 5% fetal bovine serum (FBS). The cell suspensions were vortexed thoroughly and incubated at 4 °C for 1 h in the dark. After incubation, the cells were washed three times by centrifugation at 1200 rpm for 10 min each. Secondary antibodies, Goat anti-rabbit IgG Alexa Fluor 488 and Goat anti-rat IgG Alexa Fluor 647 (Abcam, UK), were diluted 1:1000 in staining buffer containing 5% FBS and added directly to the flow cytometry tubes containing the cell pellets. The samples were vortexed and incubated for 1 h at 4 °C in the dark. Following incubation, the cells were washed three additional times, resuspended in DPBS, and analyzed using a flow cytometer (BD Accuri™ C6, Milpitas, CA, USA). In the flow cytometry analysis, Alexa Fluor 488 (Thermo Fisher Scientific, Wartham, MA, USA) dye, excited by a 488 nm laser line and detected in the FL1 channel, and Alexa Fluor 647 (Thermo Fisher Scientific, Wartham, MA, USA) dye, excited by a 640 nm laser line and detected in the FL4 channel, were used. A total of 15,000 events were acquired for each circulating blood sample.

### 2.5. Localized NETs, Neutrophils, Platelets, and Endothelial Cells in Thrombotic Femoral Artery Assessed by Flow Cytometry

Femoral artery samples were homogenized, and the resulting tissue suspensions were transferred into 5 mL conical tubes. To facilitate enzymatic digestion, 5 mL of collagenase solution (Sigma-Aldrich, Burlington, MA, USA) was added to each tube, followed by incubation in a 37 °C water bath for 1 h. After digestion, the samples were passed through a cell strainer (SPL Lifesciences, Pocheon, Republic of Korea), then washed twice with 5 mL of PBS and centrifuged at 1600 rpm for 10 min per wash. The resulting cell pellets were fixed with 300 µL of Cytofix (BD Biosciences, USA) for 15 min, gently resuspended in 5 mL of DPBS, and permeabilized using 1× Perm/Wash buffer, followed by centrifugation at 1600 rpm for 10 min. For immunostaining, the following primary antibodies were used: anti-myeloperoxidase (MPO) and anti-Ly6B.2 antibodies (Abcam, UK) for identifying NETs and neutrophils, respectively; anti-CD31 antibody (Abcam, UK) for platelet detection; and anti-CD34 antibody (Abcam, UK) as a marker of vascular endothelial cells. Anti-MPO and anti-Ly6B.2 antibodies were diluted 1:500, and anti-cluster of differentiation (CD)31 and anti-CD34 antibodies were diluted 1:400 in staining buffer containing 5% fetal bovine serum (FBS). The samples were vortexed thoroughly and incubated at 4 °C in the dark for 1 h. After primary antibody incubation, the samples were washed three times by centrifugation at 1200 rpm for 10 min each and resuspended in staining buffer with 5% FBS. Secondary antibodies, Goat anti-rabbit IgG Alexa Fluor 488 and Goat anti-rat IgG Alexa Fluor 647 (Abcam, UK), were diluted 1:1000 in staining buffer and added directly to the flow cytometry tubes containing the stained cells. The samples were vortexed and incubated for 1 h at 4 °C in the dark. Following incubation, the cells were washed three times by centrifugation at 500× *g* for 10 min, resuspended in PBS, and analyzed using a flow cytometer. In the flow cytometry analysis, Alexa Fluor 488 and Alexa Fluor 647 were used, and a total of 15,000 events were acquired for each circulating blood sample.

### 2.6. Localized NETs, Platelets, Thrombus, Neutrophils, and Fibrinogen in Thrombotic Femoral Artery Assessed by Immunohistofluorescence Staining

Femoral artery tissues were fixed overnight in 4% paraformaldehyde (PFA) at 4 °C. After fixation, tissues were transferred to a 15 mL conical tube containing 70% ethanol and stored at 4 °C for two days. The tissues were then trimmed to an appropriate size and placed into paraffin cassettes. The cassettes were immersed in a beaker and rinsed under running tap water overnight. Paraffin infiltration was performed overnight using an automated tissue processor (Leica Biosystems, Nussloch, Germany). Embedding was conducted using an embedding station (Leica Biosystems, Germany). The tissues were vertically oriented in embedding molds filled with molten paraffin, and the paraffin cassettes were placed on top. The molds were rapidly solidified on a cold plate for 15 min and further stabilized at −20 °C for 60 min. The resulting paraffin blocks were removed from the molds and placed on ice. Tissue sections were cut at 8 µm thickness using a microtome (Leica Biosystems, Germany). The sections were floated on 40% ethanol, transferred to a warm water bath set at 38 °C, and mounted onto glass slides. The slides were dried at 43 °C for approximately 1 h. To melt the paraffin, the slides were heated at 56 °C on a hotplate for 1 h, followed by deparaffinization in xylene for 20 min. Rehydration was carried out sequentially in graded ethanol solutions: 100% ethanol (two changes, 10 min each), 90% ethanol (10 min), 80% ethanol (10 min), and 70% ethanol (10 min). Antigen retrieval was performed by microwaving 1× citrate buffer (Sigma-Aldrich, USA) for 20 min, followed by the incubation of the slides in the pre-warmed buffer for 15 min. To block nonspecific binding, the slides were incubated in a humidified chamber with 300 µL of 5% goat serum per slide for 1 h at room temperature. After blocking, the slides were washed three times with 1× PBS (5 min each), and excess buffer was removed. Primary antibodies used for staining included anti-MPO antibody (NET marker), anti-CD31 antibody (platelet marker), fluorescein isothiocyanate (FITC)-conjugated anti-Ly6B.2 antibody (neutrophil marker), D-dimer polyclonal antibody (marker of thrombus breakdown; Bioss, Woburn, MA, USA), and fibrinogen α-chain antibody (fibrinogen marker; Bioss, USA). All primary antibodies were diluted 1:400 in 5% goat serum, and 100 µL of each diluted antibody was applied to the tissue sections. The slides were incubated for 1 h at room temperature in the dark, followed by three washes with 1× PBS (3 min each). For secondary antibody labeling, Alexa Fluor 488-conjugated anti-rat IgG and Alexa Fluor 647-conjugated anti-rabbit IgG (Abcam, UK) were diluted 1:500 in 5% goat serum. Each slide received 100 µL of the secondary antibody solution and was incubated for 1 h at room temperature in the dark. The slides were then washed three times with 1× PBS (3 min each), and excess buffer was removed. The nuclei were stained with 4′,6-diamidino-2-phenylindole (DAPI; Invitrogen, USA) diluted 1:2000 in distilled water for 5 min. After nuclear staining, the slides were washed three additional times with 1× PBS, and excess liquid was removed. The slides were dehydrated through an ethanol gradient (70%, 80%, 90%, and 100%; 5 min each), followed by immersion in xylene for 5 min. Finally, antifade mounting medium (Vector Laboratories, Newark, CA, USA) was applied to the stained sections, and coverslips were placed on top. The slides were analyzed using immunofluorescence microscopy.

### 2.7. Localized Fibrinogen, Neutrophils, and Platelets in Thrombotic Femoral Artery Assessed by Western Blot Analysis

Femoral artery tissue samples stored in microtubes were weighed and minced into small fragments. The tissues were placed into microtubes containing PBS at a concentration of 100 mg/mL and homogenized on ice. The homogenates were centrifuged at 12,000× *g* for 15 min at 4 °C. The resulting supernatants were carefully transferred to fresh microtubes and stored at −80 °C until further analysis. Protein concentrations were determined using a bicinchoninic acid (BCA) protein assay kit (Thermo Fisher Scientific, USA). Eight BCA standards were prepared, and 25 µL of each standard or sample was added to 200 µL of BCA working reagent in a 96-well plate. The plates were incubated at 37 °C for 30 min, and absorbance was measured at 562 nm using a microplate reader (Thermo Fisher Scientific, USA). Based on the determined protein concentrations, the samples were diluted with distilled water and mixed with 5× SDS-PAGE loading buffer (NZYTech, Lisbon, Portugal). After vortexing and brief centrifugation, the samples were denatured by heating at 100 °C for 5 min, cooled on ice for 3 min, vortexed again, and centrifuged at 12,000× *g* for 5 min at 4 °C. Protein electrophoresis was carried out using 10% Mini-PROTEAN TGX gels (Bio-Rad, Hercules, CA, USA). A total of 30 µL of each sample and a PageRuler™ prestained protein ladder (Thermo Fisher Scientific, USA) were loaded into the gel wells. Electrophoresis was conducted at 60 V for 30 min for stacking, followed by 120 V for approximately 2 h for protein separation using a PowerPac HC power supply (Bio-Rad, USA). Following electrophoresis, the proteins were transferred onto Immuno-Blot^®^ polyvinylidene difluoride (PVDF) membranes (Bio-Rad, USA). The membranes and filter papers were pre-activated in methanol for 3 min and equilibrated in cold transfer buffer. Protein transfer was performed at 80 V for 150 min in a cold environment using ice packs. After transfer, the membranes were washed three times with 1× Tris-buffered saline containing 0.1% Tween-20 (TBS-T; Biosesang, Yongin, Republic of Korea) for 15 min each. The membranes were then blocked with 5% bovine serum albumin (BSA; Santa Cruz Biotechnology, USA) in TBS-T for 2 h at room temperature to prevent nonspecific binding. Primary antibodies—including anti-Ly6B.2, anti-fibrinogen α-chain (Bioss, USA), and anti-CD31 polyclonal antibody (Invitrogen, USA)—were diluted 1:300 in 5% BSA and incubated with the membranes overnight at 4 °C. On the next day, the membranes were washed three times with TBS-T for 15 min each. Secondary antibodies, horseradish peroxidase (HRP)-conjugated goat anti-rabbit IgG (H+L) and HRP-conjugated goat anti-rat IgG (Abcam, UK), were diluted 1:3000 in 5% BSA and incubated with the membranes for 2 h at room temperature. The membranes were again washed three times with TBS-T for 15 min each. Protein bands were visualized using Pierce™ ECL Western blotting Substrate (Thermo Fisher Scientific, USA) and incubated for 3 min. Chemiluminescent signals were detected using the iBright CL1000 imaging system (Thermo Fisher Scientific, USA), with exposure times optimized per target protein. The detected bands were quantified and statistically analyzed.

### 2.8. Statistical Analysis

Statistical analyses were performed using the Mann–Whitney U test to assess differences between the Control group and the TXA 5, 10, 20, 30 groups. Data are presented as median (interquartile range), and a *p* < 0.05 was considered statistically significant. All statistical analyses were performed using Prism software (GraphPad Software, version 8.0.1.244, USA).

## 3. Results

**Figure 1:**
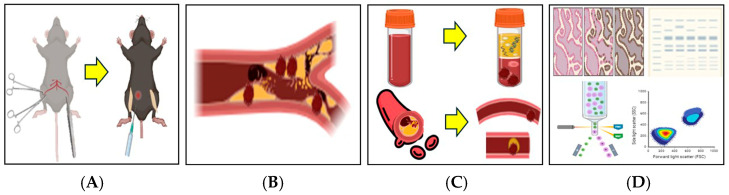
Schematic illustration of the 2.5% FeCl_3_-induced thrombosis mouse model. A thrombosis model was established by applying 2.5% FeCl_3_-soaked filter paper to the femoral artery following a surgical incision in the mouse thigh. (**A**) Bilateral thigh incisions were made, and filter paper soaked in 2.5% FeCl_3_ was applied directly to the exposed femoral artery. Tranexamic acid (TXA) was administered intraperitoneally at the time of FeCl_3_ application. (**B**) Vascular thrombosis was induced via chemical injury with 2.5% FeCl_3_. (**C**) Following treatment, blood samples and femoral artery tissues were collected for thrombus analysis. (**D**) Thrombus induction was confirmed and further evaluated using flow cytometry, immunohistofluorescence staining, and Western blot analysis. This illustration was created in BioRender. J.-W.S. (2025). https://BioRender.com/0a6y4yp, accessed on 11 April 2025.

**Figure 2:**
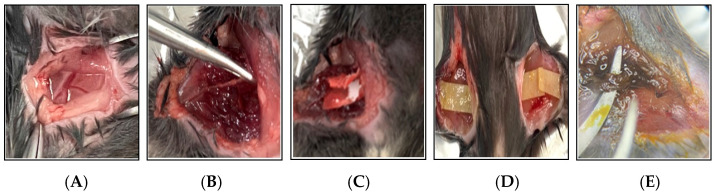
Photographic documentation of the 2.5% FeCl_3_-induced thrombosis mouse model. This figure illustrates the key procedural steps involved in the induction of thrombosis using 2.5% FeCl_3_ in a mouse model. (**A**) A surgical incision is made in the thigh to expose the femoral artery. (**B**) The femoral artery is carefully dissected and isolated from the surrounding tissues using forceps. (**C**) A strip of filter paper soaked in 2.5% FeCl_3_ solution is applied directly to the exposed artery. (**D**) To ensure consistent chemical injury, the FeCl_3_-soaked filter paper is replaced every 10 min over a 30 min period. (**E**) Vascular injury and thrombus formation are evident following 30 min of FeCl_3_ exposure.

A total of 20 thrombotic mouse models were successfully established. Blood samples and thrombotic femoral artery tissues were collected for subsequent analyses, including flow cytometry, immunohistofluorescence staining, and Western blot (Figure 1 and Figure 2).

### 3.1. Circulating NETs and Neutrophils Assessed by Flow Cytometry

**Figure 3:**
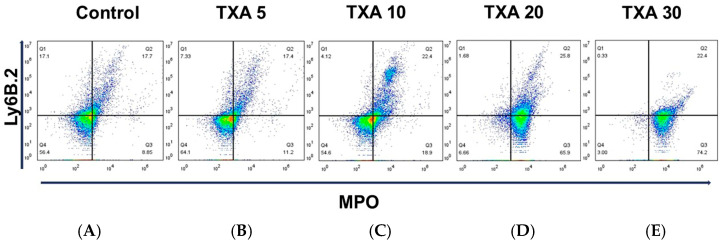
Flow cytometric analysis of circulating NETs and neutrophils. Representative flow cytometry plots showing the expression of anti-MPO antibody (NET marker) and anti-Ly6B.2 antibody (neutrophil marker) in peripheral blood across experimental groups. (**A**) Control group. (**B**) TXA 5 mg/kg group. (**C**) TXA 10 mg/kg group. (**D**) TXA 20 mg/kg group. (**E**) TXA 30 mg/kg group. Abbreviations: NETs, neutrophil extracellular traps; MPO, myeloperoxidase; TXA, tranexamic acid.

**Figure 4:**
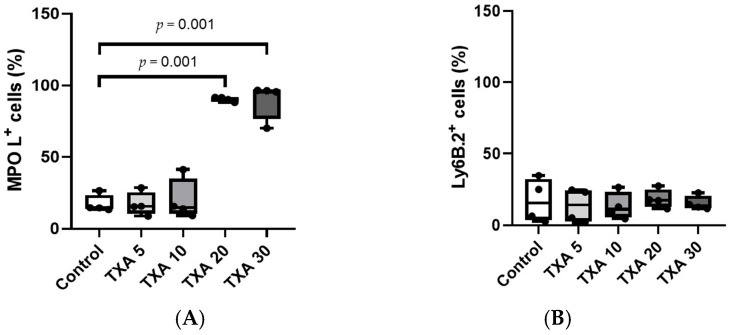
Quantification of circulating NETs and neutrophils. Statistical analysis of flow cytometry data showing the expression levels of anti-MPO antibody (NET marker) and anti-Ly6B.2 antibody (neutrophil marker) in the Control, TXA 5, TXA 10, TXA 20, and TXA 30 groups. (**A**) The expressions of anti-MPO antibody across groups. (**B**) The expressions of anti-Ly6B.2 antibody across groups. Abbreviations: NETs, neutrophil extracellular traps; MPO, myeloperoxidase; TXA, tranexamic acid.

The expressions of circulating anti-MPO antibody and anti-Ly6B.2 antibody were analyzed across the Control, TXA 5, TXA 10, TXA 20, and TXA 30 groups using flow cytometry (Figure 3). There was no significant difference in anti-MPO antibody expression between the Control group and the TXA 5 or TXA 10 groups. However, expression levels were significantly elevated in both the TXA 20 and TXA 30 groups compared to the Control group (90.9 (1.9)% vs. 14.7 (3.4)%, *p* = 0.001; 96.0 (7.1)% vs. 14.7 (3.4)%, *p* = 0.001, respectively) (Figure 4A). The expression of anti-Ly6B.2 antibody, a neutrophil marker, was comparable across all groups, with no statistically significant differences observed (Figure 4B).

### 3.2. Localized NETs and Neutrophils in Thrombotic Femoral Artery Assessed by Flow Cytometry

**Figure 5:**
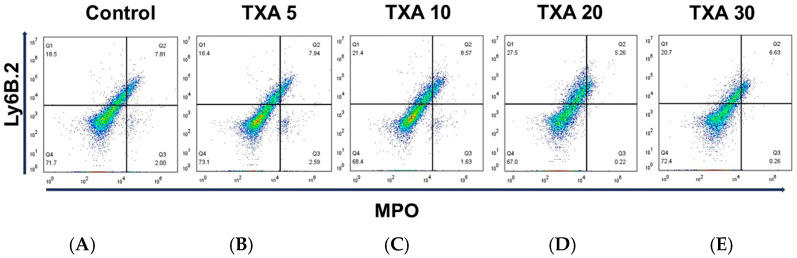
Flow cytometric analysis of localized NETs and neutrophils in thrombotic femoral artery. Representative flow cytometry plots showing the expression of anti-MPO antibody (NET marker) and anti-Ly6B.2 antibody (neutrophil marker) in thrombotic femoral artery tissue across experimental groups. (**A**) Control group. (**B**) TXA 5 mg/kg group. (**C**) TXA 10 mg/kg group. (**D**) TXA 20 mg/kg group. (**E**) TXA 30 mg/kg group. Abbreviations: NETs, neutrophil extracellular traps; MPO, myeloperoxidase; TXA, tranexamic acid.

**Figure 6:**
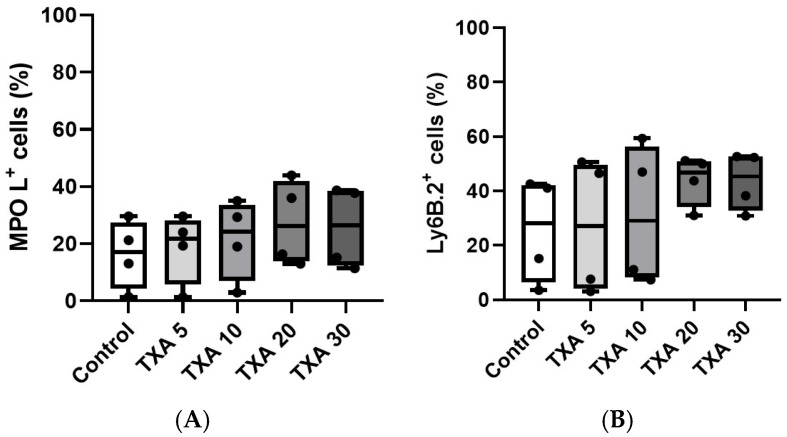
Quantification of localized NETs and neutrophils in thrombotic femoral artery. Statistical analysis of flow cytometry data showing the expression of anti-MPO antibody (NET marker) and anti-Ly6B.2 antibody (neutrophil marker) in femoral artery tissues across the Control, TXA 5, TXA 10, TXA 20, and TXA 30 groups. (**A**) The expressions of anti-MPO antibody in each group. (**B**) The expressions of anti-Ly6B.2 antibody in each group. Abbreviations: NETs, neutrophil extracellular traps; MPO, myeloperoxidase; TXA, tranexamic acid.

The expressions of localized anti-MPO antibody and anti-Ly6B.2 antibody in the thrombotic femoral artery were analyzed across the Control, TXA 5, TXA 10, TXA 20, and TXA 30 groups using flow cytometry (Figure 5). There were no significant differences in the expression levels of localized anti-MPO antibody or anti-Ly6B.2 antibody among the groups (Figure 6A,B).

### 3.3. Localized Platelets and Endothelial Cells in Thrombotic Femoral Artery Assessed by Flow Cytometry

**Figure 7:**
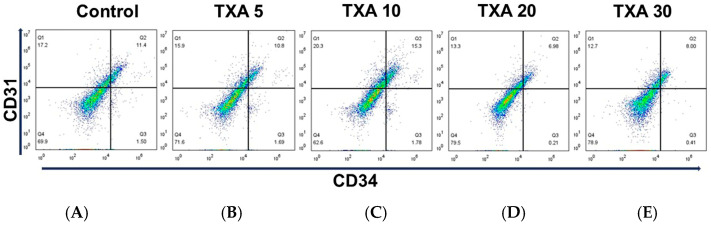
Flow cytometric analysis of localized platelets and endothelial cells in thrombotic femoral artery. Representative flow cytometry plots showing the expression of anti-CD31 antibody (activated platelet marker) and anti-CD34 antibody (vascular endothelial cell marker) in femoral artery tissues across experimental groups. (**A**) Control group. (**B**) TXA 5 mg/kg group. (**C**) TXA 10 mg/kg group. (**D**) TXA 20 mg/kg group. (**E**) TXA 30 mg/kg group. Abbreviations: CD, cluster of differentiation; TXA, tranexamic acid.

**Figure 8:**
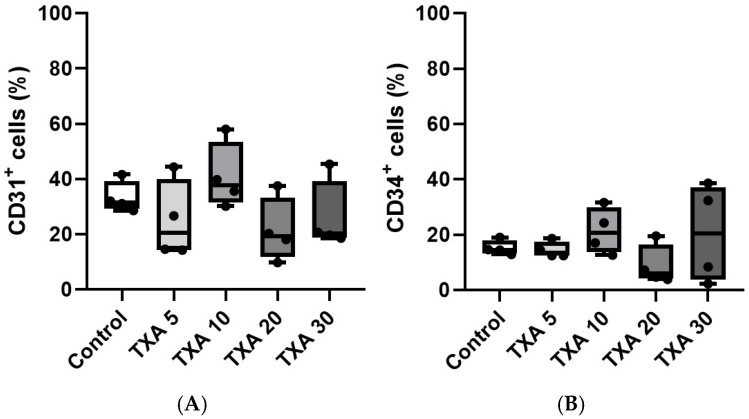
Quantification of localized platelets and endothelial cells in thrombotic femoral artery. Statistical analysis of flow cytometry data showing the expression of anti-CD31 antibody (activated platelet marker) and anti-CD34 antibody (vascular endothelial cell marker) in femoral artery tissues from the Control, TXA 5, TXA 10, TXA 20, and TXA 30 groups. (**A**) The expressions of anti-CD31 antibody in each group. (**B**) The expressions of anti-CD34 antibody in each group. Abbreviations: CD, cluster of differentiation; TXA, tranexamic acid.

The expressions of localized anti-CD31 and CD34 antibodies in the thrombotic femoral artery were assessed in the Control, TXA 5, TXA 10, TXA 20, and TXA 30 groups using flow cytometry (Figure 7). There were no significant differences in the expression of CD31 or CD34 among the groups (Figure 8A,B).

### 3.4. Localized NETs and Platelets in Thrombotic Femoral Artery Assessed by Immunohistofluorescence Staining

**Figure 9:**
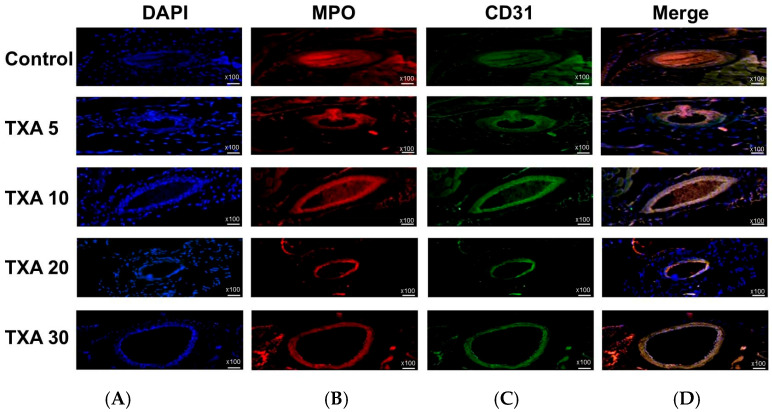
Immunofluorescence staining of localized NETs and platelets in thrombotic femoral artery. Immunofluorescence staining was performed to assess the expression of anti-MPO antibody (NET marker) and anti-CD31 antibody (platelet marker) in thrombotic femoral artery tissues from the Control, TXA 5, TXA 10, TXA 20, and TXA 30 groups. (**A**) DAPI staining showing nuclear localization in each group. (**B**) Immunostaining of anti-MPO antibody in each group. (**C**) Immunostaining of anti-CD31 antibody in each group. (**D**) Merged images showing the co-localization of NETs and platelets based on anti-MPO and anti-CD31 antibody staining. Abbreviations: NETs, neutrophil extracellular traps; MPO, myeloperoxidase; CD, cluster of differentiation; TXA, tranexamic acid; DAPI, 4′,6-diamidino-2-phenylindole.

**Figure 10:**
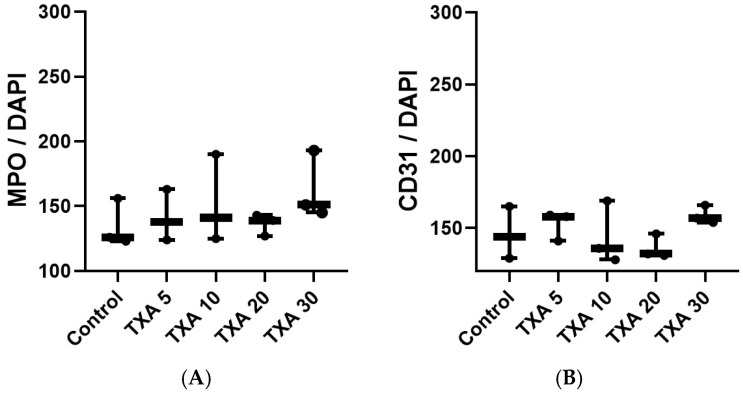
Quantification of localized NETs and platelets in thrombotic femoral artery by immunofluorescence staining. Statistical analysis of immunofluorescence staining results showing the expression of anti-MPO antibody (NET marker) and anti-CD31 antibody (platelet marker) in thrombotic femoral artery tissues from the Control, TXA 5, TXA 10, TXA 20, and TXA 30 groups. (**A**) The expressions of anti-MPO antibody in each group. (**B**) The expressions of anti-CD31 antibody in each group. Abbreviations: NETs, neutrophil extracellular traps; MPO, myeloperoxidase, CD, cluster of differentiation; TXA, tranexamic acid.

The expressions of localized anti-MPO antibody (NET marker) and anti-CD31 antibody (platelet marker) in the thrombotic femoral artery were analyzed across the Control, TXA 5, TXA 10, TXA 20, and TXA 30 groups using immunohistofluorescence staining (Figure 9). There were no significant differences in the expression of either anti-MPO or anti-CD31 antibody among the groups (Figure 10A,B).

### 3.5. Localized Thrombus and Neutrophils in Thrombotic Femoral Artery Assessed by Immunohistofluorescence Staining

**Figure 11:**
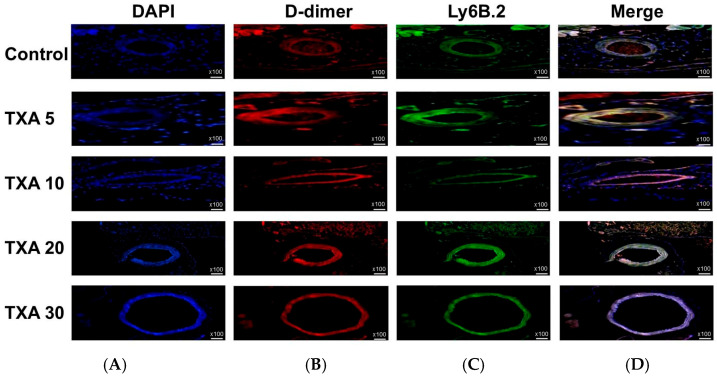
Immunofluorescence staining of localized D-dimer and neutrophils in thrombotic femoral artery. Immunofluorescence staining was performed to evaluate the expression of D-dimer polyclonal antibody (thrombus marker) and anti-Ly6B.2 antibody (neutrophil marker) in thrombotic femoral artery tissues from the Control, TXA 5, TXA 10, TXA 20, and TXA 30 groups. (**A**) DAPI staining showing nuclear localization in each group. (**B**) Immunostaining of D-dimer polyclonal antibody in each group. (**C**) Immunostaining of anti-Ly6B.2 antibody in each group. (**D**) Merged images showing the co-localization of thrombus and neutrophils based on D-dimer and Ly6B.2 staining. Abbreviations: TXA, tranexamic acid; DAPI, 4′,6-diamidino-2-phenylindole.

**Figure 12:**
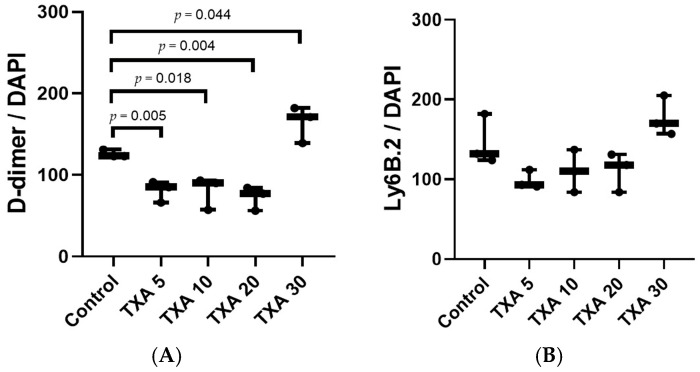
Quantification of localized thrombus and neutrophils in thrombotic femoral artery by immunofluorescence staining. Statistical analysis of immunofluorescence staining data showing the expression of D-dimer polyclonal antibody (thrombus marker) and anti-Ly6B.2 antibody (neutrophil marker) in thrombotic femoral artery tissues from the Control, TXA 5, TXA 10, TXA 20, and TXA 30 groups. (**A**) The expressions of D-dimer polyclonal antibody in each group. (**B**) The expressions of anti-Ly6B.2 antibody in each group. Abbreviations: TXA, tranexamic acid.

The expressions of localized D-dimer and anti-Ly6B.2 antibody in the thrombotic femoral artery were analyzed across the Control, TXA 5, TXA 10, TXA 20, and TXA 30 groups using immunohistofluorescence staining (Figure 11). D-dimer expression was significantly reduced in the TXA 5, TXA 10, and TXA 20 groups compared to the Control group (85.0 (12.5)% vs. 123.0 (4.0)%, *p* = 0.005; 90.0 (18.0)% vs. 123.0 (4.0)%, *p* = 0.018; 77.0 (14.0)% vs. 123.0 (4.0)%, *p* = 0.004, respectively) (Figure 12A). On the other hand, D-dimer expression was significantly increased in the TXA 30 group compared to the Control group (171.0 (21.5)% vs. 123.0 (4.0)%, *p* = 0.044) (Figure 12A). The expression of localized anti-Ly6B.2 antibody was comparable across all groups (Figure 12B).

### 3.6. Localized Fibrinogen in Thrombotic Femoral Artery Assessed by Immunohistofluorescence Staining

**Figure 13:**
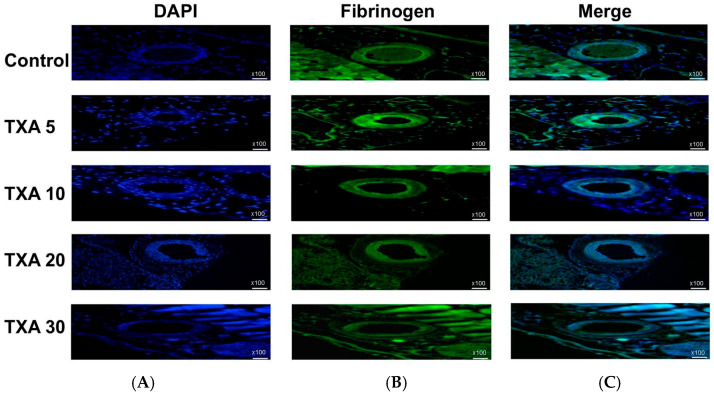
Immunofluorescence staining of localized fibrinogen in thrombotic femoral artery. Immunofluorescence staining was performed to assess the expression of fibrinogen in thrombotic femoral artery tissues from the Control, TXA 5, TXA 10, TXA 20, and TXA 30 groups. (**A**) DAPI staining showing nuclear localization in each group. (**B**) Immunostaining of fibrinogen antibody in each group. (**C**) Merged images showing the co-localization of DAPI and fibrinogen staining in each group. Abbreviations: TXA, tranexamic acid; DAPI, 4′,6-diamidino-2-phenylindole.

**Figure 14:**
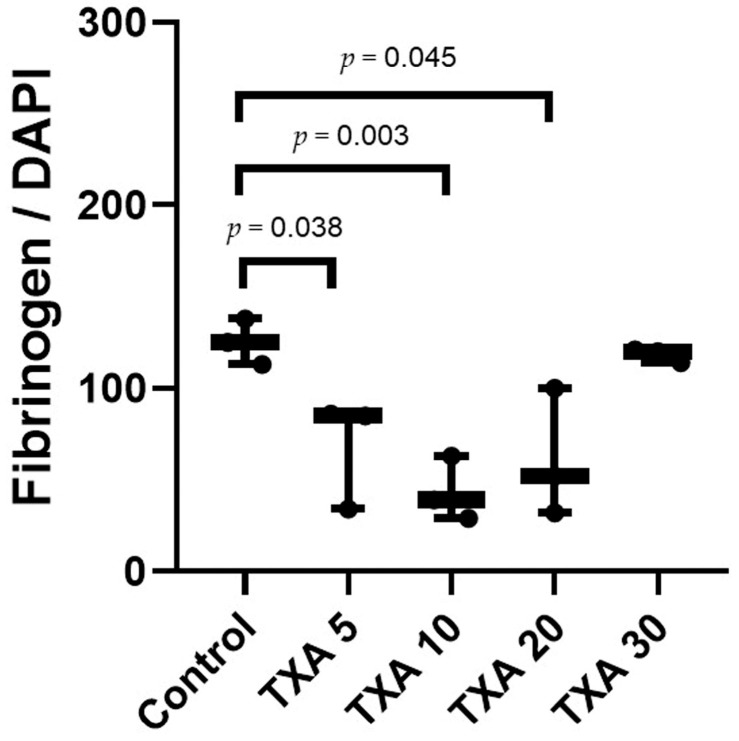
Quantification of localized fibrinogen in thrombotic femoral artery by immunofluorescence staining. Statistical analysis of immunofluorescence staining showing the expression of fibrinogen antibody in thrombotic femoral artery tissues from the Control, TXA 5, TXA 10, TXA 20, and TXA 30 groups. Abbreviations: TXA, tranexamic acid.

The expression of fibrinogen in thrombotic femoral artery tissues was evaluated in the Control, TXA 5, TXA 10, TXA 20, and TXA 30 groups using immunohistofluorescence staining (Figure 13). Fibrinogen expression was significantly lower in the TXA 5, TXA 10, and TXA 20 groups compared to the Control group (85.0 (26.0)% vs. 125.0 (12.5)%, *p* = 0.038; 39.0 (17.0)% vs. 125.0 (12.5)%, *p* = 0.003; 52.0 (34.0)% vs. 125.0 (12.5)%, *p* = 0.045, respectively) (Figure 14). However, no statistically significant difference in fibrinogen expression was observed between the TXA 30 group and the Control group (Figure 14).

### 3.7. Localized Neutrophils, Fibrinogen, and Platelets in Thrombotic Femoral Artery Assessed by Western Blot Analysis

**Figure 15:**
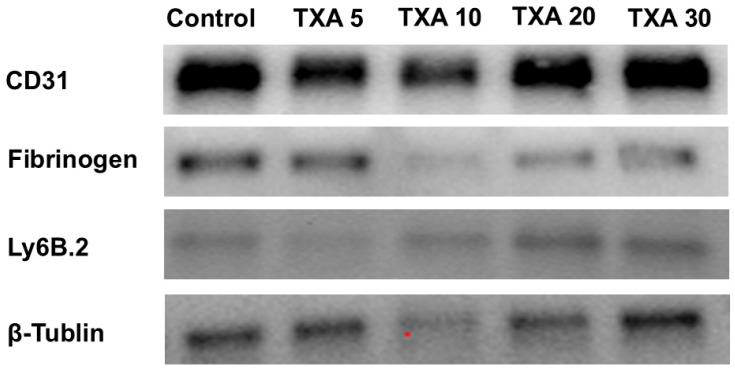
Western blot analysis of localized neutrophils, fibrinogen, and platelets in thrombotic femoral artery. Western blot analysis showing the expression of anti-Ly6B.2 antibody (neutrophil marker), fibrinogen antibody (fibrinogen marker), and anti-CD31 antibody (platelet marker) in thrombotic femoral artery tissues from the Control, TXA 5, TXA 10, TXA 20, and TXA 30 groups. Abbreviations: CD, cluster of differentiation; TXA, tranexamic acid.

**Figure 16:**
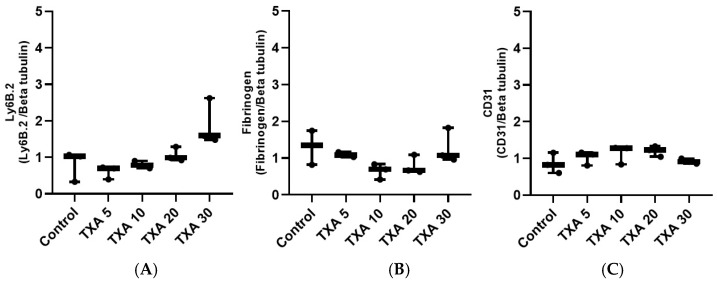
Quantification of localized neutrophils, fibrinogen, and platelets in thrombotic femoral artery by Western blot analysis. Statistical analysis of Western blot data showing the expression of anti-Ly6B.2 antibody (neutrophil marker), fibrinogen antibody, and anti-CD31 antibody (platelet marker) in thrombotic femoral artery tissues from the Control, TXA 5, TXA 10, TXA 20, and TXA 30 groups. (**A**) The expressions of anti-Ly6B.2 antibody in each group. (**B**) The expressions of fibrinogen antibody in each group. (**C**) The expressions of anti-CD31 antibody in each group. Abbreviations: CD, cluster of differentiation; TXA, tranexamic acid.

The expressions of anti-Ly6B.2 antibody, fibrinogen α-chain, and anti-CD31 antibody in thrombotic femoral artery tissues were evaluated by Western blot analysis across the Control, TXA 5, TXA 10, TXA 20, and TXA 30 groups (Figure 15). Anti-Ly6B.2 antibody expression exhibited an increasing trend with higher TXA doses. However, the differences were not statistically significant (Figure 16A). The expression levels of fibrinogen α-chain and anti-CD31 antibody were comparable across all groups (Figure 16B,C).

## 4. Discussion

This study demonstrated that thrombus formation was promoted following TXA administration at doses exceeding 30 mg/kg in a thrombosis mouse model. Additionally, high-dose TXA administration enhanced circulating NETs.

Various studies showed the beneficial effects of TXA irrespective of dose [27,28,29,30]. However, TXA administration can be seriously risky in patients with trauma, those with a history of thromboembolic disease, and critically ill patients [10,11,31,32]. In these conditions, adequate administration time and proper dosage are very important [33,34,35]. Circulating NETs were significantly elevated following TXA administration at doses of 20 mg/kg and 30 mg/kg. Additionally, fibrinogen levels were restored, and D-dimer, a protein fragment produced during fibrinolysis [36], was markedly elevated at the 30 mg/kg dose. Current evidence does not conclusively support a direct prothrombotic effect of TXA. However, TXA may induce significant inflammation by activating the complement system in injured tissues through enhanced plasmin generation in a fibrin-independent manner [37]. Furthermore, localized intravascular pathological thrombosis leads to blood flow instability within the vessel and triggers coagulation system dysregulation, resulting in endothelial cell activation, leukocyte aggregation, and the amplification of inflammatory signaling, which in turn promotes neutrophil chemotaxis [38,39]. The present study suggested a possible role of high-dose TXA in inflammation-associated thrombosis by increasing circulating NETs.

Previous studies have demonstrated that neutrophils, platelets, and endothelial cells interact with localized NETs to contribute to thrombus formation [40,41]. However, our study highlights the potential role of circulating NETs as key drivers of thrombus formation in the context of high-dose TXA administration. This suggests a shift in the thrombotic mechanism from primarily localized cell-to-cell interactions to systemic NET-mediated enhancement at higher TXA concentrations. Taken together, our findings suggest that the antifibrinolytic effect of TXA may be insufficient to promote thrombosis at doses below 20 mg/kg. In contrast, at doses ≥30 mg/kg, TXA appears to exert a more potent prothrombotic effect, likely mediated through enhanced NET formation and the restoration of fibrinogen, ultimately facilitating localized thrombosis.

NETs act as potent triggers of thrombosis and are known to be elevated in various pathological conditions such as vasculitis, chronic deep vein thrombosis, and ischemia [13,42,43,44]. Under these conditions, high-dose TXA administration may further exacerbate thrombus formation. As a result, several studies have proposed targeting NET release as a therapeutic strategy to attenuate thrombosis [45,46,47]. However, our findings indicate that the extent of localized NET release in thrombotic tissue was not significantly affected by TXA dosage. In contrast, circulating NETs were significantly increased at higher TXA doses. Based on this observation, we propose that inhibiting circulating NETs, rather than localized NET release, may be a more effective therapeutic approach to mitigate thrombus formation in patients receiving high-dose TXA.

In the present study, no significant increase in circulating NETs, neutrophils, or platelets was observed following TXA administration at doses of 5 mg/kg or 10 mg/kg. Similarly, doses of 20 mg/kg to 30 mg/kg did not result in elevated levels of circulating neutrophils, platelets, or endothelial cells. Localized levels of NETs, neutrophils, platelets, and endothelial cells in thrombotic tissue also remained unchanged across all TXA doses. Notably, both fibrinogen and D-dimer levels in thrombotic tissue were simultaneously suppressed with TXA doses ranging from 5 mg/kg to 20 mg/kg. This dual suppression suggests a reduced capacity for fibrin formation and subsequent clot development, indicating that thrombus formation may be insufficient at TXA doses below 20 mg/kg. Varma et al. demonstrated that leukocytes not only contribute to thrombus formation but also regulate thrombus size by activating fibrinolytic mediators [48]. Additionally, effective thrombus formation requires coordinated interaction among neutrophils, platelets, and endothelial cells [3]. In this context, the lack of increase in circulating NETs and neutrophils, the significant reduction in fibrinogen within thrombotic tissues, and the stable expression of localized CD31 and CD34, markers of platelet–endothelial interaction [49,50,51], suggest minimal cell-to-cell interaction necessary for thrombus development. These findings collectively support the notion that TXA doses below 20 mg/kg are unlikely to sufficiently promote thrombus formation. Based on the findings of this study, clinicians should be aware that high-dose TXA administration may carry a potential risk of promoting thrombus formation in thrombotic conditions, primarily through the enhancement of circulating NETs.

There are several limitations to the present study. First, we did not evaluate additional NET-associated components such as extracellular DNA or histones, which are also key markers of NETs formation [52]. However, MPO, which was assessed in this study, is a well-established marker closely associated with NETs [46]. Second, the number of thrombotic mouse models used was relatively small, which may limit the generalizability of the findings. Third, TXA was administered to mice via intraperitoneal injection in the present study, whereas it is typically administered orally or intravenously in clinical settings. Additionally, the dosage conversion of TXA using allometric scaling may vary depending on the formula applied or the selected reference point, and therefore may not be entirely accurate. Therefore, direct comparisons between the experimental model and clinical practice have inherent limitations. Moreover, genetic and physiological differences between animals and humans, including disparities in drug metabolism pathways, immune responses, and coagulation systems, should be taken into account. Therefore, although TXA was observed to increase circulating NETs and promote thrombus formation in the thrombosis mouse model used in the present study, these findings alone are insufficient to directly extrapolate the same effects to humans. Therefore, future large-scale in vivo studies and clinical trials are warranted to further clarify the effects of TXA administration on NETs and thrombus formation.

## 5. Conclusions

In conclusion, this study demonstrated that localized thrombus formation may be promoted by high-dose TXA administration, underscoring the importance of careful dosage selection in thrombotic conditions. Based on our findings, high-dose TXA should be used with caution in patients with a history of thrombosis or embolism. If TXA administration is necessary, a lower dose may be more appropriate to minimize the risk of thrombus formation.

## Data Availability

The data presented in this study are available on request from the corresponding author due to restrictions imposed by the Institutional Animal Care and Use Committee, which approved the study protocol.

## Abbreviations

The following abbreviations are used in this manuscript:

TXA	Tranexamic acid
NETs	Neutrophil extracellular traps
IACUC	Institutional Animal Care and Use Committee
FeCl_3_	Ferric chloride
PBMC	Peripheral blood mononuclear cell
DPBS	Dulbecco’s phosphate-buffered saline
RBCs	Red blood cells
MPO	Myeloperoxidase
FBS	Fetal bovine serum
CD	Cluster of differentiation
PFA	Paraformaldehyde
FITC	Fluorescein isothiocyanate
DAPI	4′,6-Diamidino-2-Phenylindole
PVDF	Polyvinylidene difluoride
TBS	Tris-buffered saline
BSA	Bovine serum albumin
PBS	Phosphate-buffered saline
HRP	Horseradish peroxidase
